# Prevalence and Mechanisms of Itch in Chronic Wounds: A Narrative Review

**DOI:** 10.3390/jcm14092877

**Published:** 2025-04-22

**Authors:** Marieta Papanikolaou, Julia Paul, Leigh A. Nattkemper, Robert S. Kirsner, Gil Yosipovitch

**Affiliations:** 1 Department of Dermatology, Miami Itch Center, University of Miami Miller School of Medicine, Miami, FL 33136, USA; lxn202@med.miami.edu (L.A.N.); gyosipovitch@med.miami.edu (G.Y.); 2 School of Nursing, Oakland University, Rochester, MI 48309, USA; jcpaul3288@gmail.com; 3 Dr. Philip Frost Department of Dermatology and Cutaneous Surgery, University of Miami Miller School of Medicine, Miami, FL 33136, USA; rkirsner@med.miami.edu

**Keywords:** itch, pruritus, wound healing, wound repair, ulcer, burn

## Abstract

Itch is a commonly experienced problem by individuals with chronic wounds and greatly compromises their quality of life. Scratching can further hinder the wound healing process. Despite this being a clinically recognized issue, our knowledge of its exact prevalence in chronic wounds of different types and the molecular mechanisms driving it is limited. The multifactorial nature of wound itch makes its characterization particularly challenging. The present review is based on a thorough PubMed search, and it aims to provide an overview of existing evidence on the epidemiology, impact, and pathophysiology of wound itch, along with general recommendations on its management. Importantly, our work highlights the merit of screening chronic wound patients for associated pruritus and incorporating anti-itch measures in mainstream wound care.

## 1. Introduction

The term wound encompasses any disruption in the integrity of skin [1]. Wounds can be iatrogenic, for example after surgery, or they can arise secondary to vascular, neuropathic, traumatic, inflammatory, or pressure-related etiologies, and in association with malignancy [2]. Chronic wounds do not progress through a normal and timely sequence of repair [3], which is generally considered to be between 4 and 12 weeks [4]. Poorly healing wounds can have a major impact on quality of life (QoL) [5,6].

Itch is experienced by patients during both normal and impaired wound healing [7], and in association with wounds of various etiologies [8,9]. Importantly, itch has been identified as a key patient-reported outcome by an international wound expert panel [10]. However, wound-related itch is underrepresented in the peer-reviewed literature. No studies have been conducted to date to assess how the prevalence and characteristics of itch differ depending on wound type and stage, and our understanding of the mechanisms that underpin it is limited. Treatment of wound itch is therefore largely supportive and unsatisfactory [11].

The present work summarizes current knowledge on the prevalence, impact, and potential mechanisms of itch associated with chronic wounds, and it aims to raise awareness of the importance of addressing itch as part of routine wound care.

Itch associated with iatrogenic wounds, acquired blistering disorders, hypertrophic scars, or keloids is not covered in the present review.

## 2. Epidemiology and Impact of Wound-Associated Itch

The global prevalence of chronic wounds has been estimated at 2.21 per 1000 population [12]. In the United States, a retrospective review of Medicare claims reported a chronic wound prevalence of 16%, with a rising trend between 2014 and 2019, and associated expenditures exceeding $22 billion annually [13]. Patients with pruritus have been estimated to have higher annual healthcare costs by almost $5000 compared with patients without pruritus, including after controlling for comorbidities and sociodemographic factors. However, strikingly few studies have investigated the prevalence of itch in chronic wounds, and our search did not identify data on its associated costs. This aspect of chronic wounds is therefore poorly quantified.

Itch can be experienced within and around the wound site [7]. A cross-sectional study investigating itch in a cohort of 200 consecutive patients attending a wound care clinic reported an overall prevalence of 28%, with a mean itch numeric rating scale (NRS) severity score of 5.59, reflecting moderate-severe itch [14]. It is worth noting that patients with a rash in the area of the wound and/or a pruritic skin condition involving more than 20% of the body surface were excluded. Wound-related itch was highest in wounds of venous etiology, at 45.2%, and lowest in diabetic wounds, at 16.1%. Notably, the mean itch NRS score (average itch intensity in the last 24 h) of 5.59 was higher than patients’ worst bodily pain NRS score (worst pain intensity in the last 24 h) mean of 4.25. Positive associations were noted between itch and wound size, as well as patient age [2,14]. A weak but statistically significant positive correlation was also noted between patients’ itch and pain scores [14]. Another study involving 531 patients with over 2000 wounds of various etiologies reported itch in 5% of participants making it one of their seven commonest complaints, with an average itch NRS severity score of 2.75 [15]. However, patients with alternative causes for itch were not excluded in this particular study, hence the possibility of correlation rather than causation cannot be excluded.

Scratching can cause tissue damage and lead to infection [16], thus further hindering healing or even leading to new wounds, with detrimental results for patients’ QoL [10,17,18,19]. Limited data are available on the exact impact of wound-related pruritus on QoL; however, its magnitude is reflected by negative descriptors commonly used by patients [20]. Alleviation of itch should therefore be incorporated into wound care practice, to prevent wound deterioration and improve QoL in affected individuals.

## 3. Physiology of Wound Healing

Wound healing is an intricate orchestration of interactions between keratinocytes, platelets, fibroblasts, endothelial and immune cells, and the extracellular matrix (ECM). It unravels in four main stages: hemostasis, inflammation, proliferation (tissue formation), and remodeling (scar formation), which are overlapping [21,22]. This sequence of events can take anywhere between a few months to a year [23], and is influenced by several systemic factors, including the individual’s age [24], smoking status [25], nutritional status [26,27], comorbidities [28,29], or regular medications [30], as well as local factors such as the presence of infection and biofilm formation [31].

Numerous cells and molecules have been identified as key mediators of wound healing. The first stage of healing (hemostasis) starts immediately after injury, with platelets being exposed to the ECM secondary to vascular disruption [22,32]. They thus adhere to damaged vessels and release their granules, initiating a coagulation cascade that leads to the formation of a fibrin clot. Platelets are also known to release growth factors and chemotactic factors, attracting immune cells to the site [32,33].

Alongside platelet activation, disruption of the epidermis triggers keratinocytes to release proinflammatory cytokines, including interleukin 1 (IL-1) and tumor necrosis factor-alpha (TNF-α), which in turn stimulate both keratinocytes and fibroblasts to produce mediators of inflammation and proliferation [34]. Gamma delta T cell receptor-bearing dendritic epidermal T cells (DETCs) respond to antigens expressed by damaged or stressed keratinocytes by producing keratinocyte growth factors (KGF), insulin growth factor 1 (IGF-1), and chemokines in an IL-15 dependent manner [35,36,37]. During the inflammatory phase, chemokines produced by platelets, DETCs, and keratinocytes attract blood macrophages and neutrophils to the injured site. One of the main functions of leukocytes is the production of reactive oxygen species (ROS) and proteases to clear the wound bed of foreign bodies, dead cells, and pathogens [33]. Activated neutrophils are also the main source of inflammatory molecules, which mediate the recruitment of more leukocytes, macrophages, and T cells to the site [38]. Macrophages are a major cell type during the inflammatory phase but are also key for the transition to the proliferative stage of wound healing. Both tissue-resident macrophages and migrating monocytes differentiating into macrophages are involved [28]. These can switch between pro-inflammatory and anti-inflammatory phenotypes under the influence of fibroblast-derived exosomes [39], promoting or inhibiting the inflammatory stage of healing, respectively [28].

Resolution of wound inflammation signals the start of the proliferative phase of healing, two days to a week from the initial injury [33]. Keratinocytes and fibroblasts migrate to the site and proliferate under the influence of growth factors, leading to granulation tissue formation. This is accompanied by the production of new ECM, angiogenesis, and differentiation of fibroblasts to myofibroblasts. Periostin, a multifaceted ECM protein and mediator of fibrosis has been shown to play an important role in the differentiation of fibroblasts to myofibroblasts [40]. The latter generate contractile forces and accelerate tissue repair [41]. Notably, recent evidence also suggests that Schwann cells, the glial cells of the peripheral nervous system release important molecules during this stage, promoting axonal regeneration, as well as influencing the migration and proliferation of other cell populations [42].

During remodeling, the fourth and last stage of healing, fibroblasts proliferate further and drive the remodeling of the ECM alongside re-epithelialization of the wound. Type III collagen is degraded, and type I collagen is synthesized in its place. Proteases are major mediators of this process [43] and are derived from several cellular sources, including activated keratinocytes, fibroblasts, endothelial cells, invading leukocytes, and macrophages [44]. The balance between activation and inhibition of matrix metalloproteinases is crucial for healthy tissue remodeling and eventually healthy scar formation [45].

Special mention should finally be made to neuropeptides such as calcitonin gene-related peptide (CGRP), substance P (SP), and nerve growth factor (NGF), released by both resident cells and cutaneous nerve endings in the skin, in response to trauma or inflammatory molecules [46]. There is increasing evidence that these play important roles across several stages of wound healing, including inflammation, proliferation, as well as wound remodeling [47,48,49].

## 4. Pathophysiology of Chronic Wounds

Inflammation itself, as well as successful transition from the inflammatory to the proliferative stage of wound healing, are both crucial to successful tissue repair and scar formation. There is evidence to suggest that chronic wounds fail to progress through these stages and thus remain in a constant state of inflammation [44], characterized by impairment in keratinocytes’ ability to migrate and proliferate [50]. In line with this, the transition from a chronic to an acute inflammation state has been shown to promote wound healing [51,52,53].

Excessive unimpeded proteolytic activity is also a cardinal feature of poorly healing wounds. Persisting immune cells continue to produce proinflammatory cytokines, promoting matrix metalloproteinase (MMP) activity while downregulating the expression of MMP inhibitors [44]. Unimpeded MMP activity leads to the degradation of molecular components that are necessary for wound repair [43].

Reactive oxygen species (ROS), normally crucial for the destruction of pathogens in wounds, are overproduced in chronic wounds [54]. This can have several detrimental effects, including ROS-mediated upregulation of pro-inflammatory cytokines, induction of MMPs, direct damage to ECM proteins, and interference with fibroblast function [54].

Finally, overgrowth of bacterial components and polymicrobial infection are prominent in non-healing wounds [31,55], with evidence that these have a direct adverse effect on the healing process [56]. Bacteria can form biofilms to evade immune surveillance and multiply, thereby impeding wound healing through several mechanisms, such as the perpetuation of inflammation, inhibition of host defense, and impairment of fibroblast function [57,58,59,60]. In line with this, the level of wound bioburden has been identified as a determinant of how well a wound can heal, and reducing this is a therapeutic priority [61].

## 5. Why Are Wounds Itchy

The reasons why wounds become itchy are unknown. Pathways involved in wound pruritus are likely to differ depending on wound etiology and stage of healing, considering the major differences in the molecular profile across stages and conditions. Numerous non-histaminergic itch mediators and pathways have been identified in recent years, which can explain the lack of effectiveness of antihistamines for chronic itch, such as in the case of atopic dermatitis [62,63]. Chronic wounds differ from other causes of chronic pruritus in that the inflammatory state is combined with disturbance of normal skin architecture and increased exposure to environmental pathogens. Chronic wound itch is therefore likely multifactorial, with contributions from all these factors.

From an evolutionary standpoint, itch is thought to have evolved as a mechanism to remove irritants or harmful pathogens from the skin through scratching [64]. Notably, Liu et al. recently demonstrated that scratching enhances immune cell-mediated antibacterial skin inflammation [65]. It is possible that itch and scratching serve such roles during physiological wound healing. It is uncertain however whether such findings are transferable to chronic wounds, which represent a pathological state. Prolonged scratching in this case can exacerbate tissue damage, lead to new wounds, and increase the risk of bacterial infection, as pathogens are transferred into and around the area of disrupted skin integrity.

### 5.1. Overlap Between Mediators of Wound Healing and Itch

Several cell types and molecular mediators of wound healing are also key itch players. It is therefore plausible that mediators produced as part of the healing process activate peripheral itch receptors and hence trigger itch signals within wounds. There is a striking paucity of studies investigating the expression and effects of itch markers in wounds. Xu et al. demonstrated increased IL-31 levels in wound tissue at the peak of the itch response in mice, whereas Il31−/− mice lacked wound-induced itch responses [66]. In a different study, topical application of Emorsan^®^ Gel led to a reduction of pro-inflammatory cytokines IL-1β and TNF-α, as well as mast cell infiltration in a wound healing mouse model. These changes were associated with a significant reduction in scratch bouts [67]. Upregulation of neuropeptides in burn wounds and scars, including ubiquitin C-terminal hydrolase L1 (also known as protein gene product 9.5), SP, and CGRP—known to also be implicated in pruritus [68,69,70]—has been demonstrated by several studies, both at the clinical and preclinical stage [71]. Periostin is another mediator of interest; an ECM protein with well-known roles in wound healing [72], which has been shown to induce pruritus by binding to integrin αVβ3 on itch-transmitting DRG neurons [73]. Mediators of wound healing that are also known direct pruritogens (i.e., pruritogens that can directly activate itch-transmitting neurons), are presented in Table 1. These molecules have roles during the inflammatory, proliferative, as well as remodeling stages of wound healing; it is thus possible that they concomitantly trigger itch at any of these stages.

### 5.2. Neuronal Activity

Neuronal signals are crucial to tissue regeneration [49,150]. It has been shown that electrical stimulation of cutaneous wounds leads to accelerated healing [151], whereas this is significantly impaired in the absence of nerves [152]. Sensory abnormalities, including loss of sensation, paraesthesia, and chronic pain have commonly been reported in burn wounds [153], and these could be a result of long-term changes in cutaneous innervation of the affected sites [154]. Palanivelu et al. demonstrated that the ratio of bodies of itch-transmitting neurons to those responsible for touch, vibration, and proprioception increased in ipsilateral DRGs following burn injury in a rat model [154]. The group hypothesized that such changes in DRGs could mediate changes in cutaneous innervation over burn sites, leading to abnormal pain and itch thresholds clinically. Only one preclinical study has directly investigated the relation of skin reinnervation with itch in a rat burn wound model, but this did not show a correlation between scratching behavior and reinnervation patterns [155]. More research is required to clarify the impact of neuronal regeneration and activity on wound pruritus.

Another mechanism by which neuronal activity could be triggering itch sensation in chronic wounds is by the release of neuropeptides, small proteins capable of binding to and activating neurons, as well as immune cells. Neuropeptides are released by both sensory and autonomic nerve fibers in the skin [49]. They have increasingly recognized and versatile roles in all stages of wound healing; in particular, there is evidence to support their involvement in vasodilation, chemotaxis, regulation of the inflammatory response, re-epithelialization, angiogenesis, nerve regeneration, and eventually wound contraction and remodeling (Table 1) [49,127]. Simultaneously, neuropeptides are well-recognized mediators of itch (Table 1). In the periphery, their release by cutaneous nerve endings can enhance and perpetuate the production of pruritogens by immune cells, in a process known as neurogenic inflammation [156]. Based on existing evidence, a dual role in wound healing and itch in chronic wounds can thus be hypothesized.

### 5.3. Microbial Factors

Disruption of skin integrity exposes underlying tissues to pathogenic microorganisms, leading to microbial contamination, subsequent colonization, and in some cases, infection [29]. In zebrafish, infection with Listeria induced persistent inflammation and impaired healing, which were partially rescued by IL-1R signaling blockade and early antibiotic intervention [60].

Staphylococcus aureus and Pseudomonas aeruginosa are the most commonly isolated pathogens from human chronic wounds [31,157]. Overgrowth of Staphylococcus aureus is also a feature of atopic dermatitis, a highly pruritic skin condition [158]. In vitro, staphylococcal enterotoxin B was shown to induce leukocytes to overexpress IL-31, a key itch cytokine [159]. More recently, it was demonstrated that S. aureus-derived V8 protease induces itch via activation of PAR-1 receptors on peripheral sensory neurons [160]. In line with this, a positive association between biofilm formation and wound-related symptoms, including itching, has been demonstrated in human subjects with chronic venous ulcers [161], implying a role for microbes in wound-related itch.

### 5.4. Other Factors

Numerous other factors could be triggering or contributing to wound-related itch [162,163]. Moisture levels [10,162], wound pH [162], exudate [164], wound tension [162], topical applications [165,166], use of dressings [166] or compression [167], analgesia especially with opioids [168], contact-type reactions [169,170,171,172,173], underlying xerosis [174], pre-existing skin diseases [175], or stress [176] have been reported to influence the incidence and intensity of itch. The complex, multifactorial nature of wound pruritus thus makes its study and characterization all the more challenging.

## 6. Itch Associated with Burns

Pruritus is highly prevalent among burn sufferers, both in acute and chronic settings [177]. It has a great impact on patients’ QoL and mental well-being [178], while currently, available therapies are failing to achieve clinically significant symptomatic relief [11]. In a prospective study of adult burn survivors, 93% experienced pruritus at discharge, and this percentage remained high, at 73% two years following the initial injury [179]. Notably, over 40% of individuals 4–10 years post-burn in the study still reported pruritus in the burn, graft, or donor area. Similarly, the prevalence of pruritus in a pediatric burn survivor cohort was 93% at discharge and remained at 63% after two years [180]. Other studies have estimated the prevalence of post-burn pruritus in pediatric populations between 37–72% [181,182,183]. A trial of low-energy extracorporeal shockwave therapy in 45 adult patients with partial or full-thickness burns reported a median itch NRS severity score of 7 pre-treatment, corresponding to severe itch [184]. Burn size, injury depth, female sex, race, post-traumatic stress symptoms, and pre-existing skin disease have all been identified as predictors of itch intensity in burn survivors [175,179,181,182,185,186,187,188].

The exact pathomechanism of post-burn pruritus is unknown. Compared to normal wound healing, burn injuries trigger a pronounced and persistent inflammatory response, both at local and systemic level [189]. This is characterized by an immediate surge in neutrophils at the site of injury, followed by rises in macrophages and lymphocytes [190]. These immune infiltrates persist into what would normally be the proliferation and maturation stages of wound healing. The local reaction is accompanied by the release of pro-inflammatory cytokines into the peripheral circulation, particularly IL-6, IL-8, IL-10, and monocyte chemoattractant protein-1, in proportion to the body surface area involved [191,192]. This profound immune response could be driving or contributing to post-burn pruritus, albeit these markers have not yet been correlated to itch in burn patients.

It is plausible that mechanisms differ depending on the stage of healing (burn wound versus burn scar), as indicated by the fact that histaminergic signaling blockade becomes less effective in the late proliferative and remodeling phases [177]. Overall, the efficacy of neuropathic agents, along with their superiority to antihistamines in post-burn pruritus, points to a predominantly non-histaminergic neuropathic mechanism [177,193]. Increased expression of transient receptor potential (TRP) channels, as well as neuropeptides SP and CGRP, has indeed been demonstrated in burn scars [194,195,196].

## 7. Itch Associated with Cutaneous Ulcers

Ulcers involve the loss of tissue extending deeper than the basement membrane zone [197]. They can arise secondary to various etiologies, including vascular dysfunction, diabetes, pressure, inflammatory conditions, infections, neoplasms, or drugs, and they primarily affect the lower limbs [198]. Chronic venous insufficiency (CVI) is a leading cause of leg ulcers [199]. Ulcers secondary to CVI affect 1% of the US population and recurrence rates are high, including after surgical intervention [200].

There is a marked paucity of studies focusing on the prevalence of pruritus in patients with cutaneous ulcers, as well as variability in its reported prevalence possibly due to differences in underlying pathologies and data recording strategies. In a cross-sectional study of 50 individuals with chronic leg ulcers of various etiologies, 68% reported pruritus, with a median intensity of 3 on the NRS in the preceding 24 h. The authors noted that itch affected the wound itself, the skin surrounding it, or both [6], and increased in intensity with age. Less than half of the patients were noted to have background xerosis, with the majority not demonstrating abnormalities on examination of their remaining skin surface. Patients with pruritus had a higher mean Wound-QoL score (32.1 vs. 25.8), indicating greater impact on QoL. Similarly, in semi-structured interviews of 38 patients with venous leg ulcers, 69% reported itch beneath the bandages or around the ulcer [5]. In the case of bandages, neural sensitization secondary to chronic wound-related itch could not be excluded as an additional itch mechanism, i.e., that mechanical provocation by the bandage itself triggers or intensifies itch [201]. Two clinical trials—one of autologous platelet-rich plasma [202] and one of calcium dobesilate [203]—in 16 and 25 individuals with refractory venous ulcers reported mean pre-therapy itch scores of 2.69 and 3.55, respectively, on a 4-point grading system, reflecting moderate/severe pruritus. In a different study, trialing Sepaderm (Aalnex, Inc., Irvine, CA, USA) in 14 patients with either venous or diabetic leg ulcers reported itch at baseline in three (21%) participants [204].

It is worth noting, however, that itch is commonly reported in association with varicose veins [205], peripheral edema, stasis dermatitis, or CVI [206] in the absence of ulcers. One study excluding patients with cutaneous ulcers estimated itch prevalence in CVI at 66%, with an average intensity of 2.9 cm, as measured on a 10-cm VAS scale [206]. Itch affecting the lower limbs was reported in 45% of individuals with CVI in a different cohort, only 19% of whom had associated wounds on the legs or feet [207]. Notably, around 40% of individuals with leg ulcers have been diagnosed with stasis dermatitis [208], which is a pruritic condition. It is therefore unclear whether itch in the context of venous ulcers is driven by background CVI and/or stasis dermatitis, or the wound itself. Additionally, ulcers are overall more common in the elderly population [209], who are more prone to developing itch secondary to several other factors or comorbidities [169,210].

Similarly, pruritus in diabetic ulcers is hard to quantify in view of the high prevalence of itch in this patient population in the absence of ulceration [211,212]. Additional complicating factors include the presence of diabetic neuropathy, which could be dampening or enhancing pruritic signaling, along with susceptibility of this group to infection due to impaired antimicrobial barrier [213].

Itch can arise within skin cancers [214,215], and it is amongst the commonest problems of people with malignant fungating wounds [216,217]. It has been reported to affect 5–50% of malignant wounds [218,219]. However, a study assessing itch in skin cancer patients found no association between itch intensity and the presence of ulceration [214]. Subgroup analysis of squamous cell carcinomas in the same study on the other hand showed a trend of inverse association between itch and ulceration.

Itch has been reported in association with ulcers of other etiologies, including vasculitis [220,221], pyoderma gangrenosum with response to topical tacrolimus [222] and interleukin-23 blockade [223], and trigeminal trophic syndrome ulceration [224,225]. We could not identify reports focusing on itch in pressure ulcers; quantification of any associated pruritus could be challenging as the latter are most common in older patients, particularly in the palliative setting [226].

Finally, itch of variable degrees has been reported in infective ulcers, including cutaneous tuberculosis [227], botryomycosis [228], and cutaneous mucormycosis [229]. On the other hand, cutaneous ulcers secondary to other entities such as cutaneous leishmaniasis [230] or syphilitic chancre [231] do not tend to be itchy for reasons that are unclear.

## 8. The Special Case of Epidermolysis Bullosa

Epidermolysis bullosa (EB) encompasses a heterogeneous group of inherited blistering disorders characterized by skin frailty and repeated cycles of blistering, which can lead to extensive chronic wounds [232]. Itch is a common problem in all EB types but mostly so in junctional and dystrophic EB (DEB) [233,234]. Its reported prevalence is between 85 and 98% [233,234,235,236], with severity comparable to that of atopic eczema [236]. In-depth, semi-structured interviews of six subjects with EB of various types revealed reciprocity between itch and wounds, the constant nature of itch, and the lack of control over itch as recurrent themes, reflecting the devastating impact of EB itch on QoL [19].

Recent studies have demonstrated that Th2 upregulation contributes to EB-related itch [237,238,239], which is supported by the clinical success of dupilumab in cases of DEB [240,241]. It is uncertain whether EB-related pruritus is generated in wounds, or if this has a systemic source. Increased circulating levels of C-reactive protein (CRP), periostin, inflammatory cytokines (IL-1, IL-6, IL-31, TSLP, among others), as well as Th2 skewing of peripheral T-cells, have been reported in EB subjects [239,242,243,244,245], implying a systemic inflammatory component. Additionally, reduction of itch with pregabalin in a recent randomized, placebo-controlled study of recessive DEB favors a central neuropathic component [246]. On the other hand, healing EB wounds and their surrounding skin have been reported to be more itchy than intact skin [233,247], while a few studies have shown that itch was higher in frequency and severity in more severe forms of EB [247,248,249]. Chronic inflammation, barrier dysfunction, skin sensory nerve dysfunction, and fibrosis are some EB wound-related factors that could explain these findings [236]. The contribution of both local and systemic factors to EB itch is hence plausible.

## 9. Management of Wound-Related Itch

Wound-related itch has several detrimental sequalae, including compromise to patient QoL, increased risk of infection, damage to surrounding skin, and impaired wound healing secondary to scratching. Modifying wound care protocols to address wound-related itch effectively can help mitigate these. To date, there are no established strategies, guidelines, or FDA-approved treatments to alleviate wound itch [8]. Its complex multifactorial nature can complicate treatment. Antipruritic therapy for wounds poses some unique challenges. Application of topical agents directly to the wound reduces the risk of systemic side effects and ensures delivery of high therapeutic concentrations at the target site [250]; however, this can also cause irritation and therefore affect adherence. Additionally, necrotic tissue, slough, or exudate can interfere with the absorption of any topical agents. On the other hand, the distribution of systemic agents to wounds might be problematic due to disturbances in circulation locally.

The approach should encompass treatment of the underlying condition and measures to optimize healing [61,251], protection of the surrounding skin with barrier products and moisturizers [8], as well as specific anti-itch measures and therapies. The latter can be informed by the European Guidelines on Chronic Pruritus [252], which recommend a series of general anti-itch measures, along with a stepwise therapeutic approach ranging from moisturizers to systemic immunosuppressants.

In terms of general measures, strategies to prevent scratching are crucial to help minimize secondary skin damage. These can include keeping the surrounding skin well-hydrated with regular generous moisturization, application of soaked compresses to the skin around the wound, using a massage roller over clothing or rubbing instead of scratching, use of cool packs to relieve itching, or even psychological approaches for habit reversal [252,253,254].

Most mediators of wound healing that are also potential pruritogens in wounds (see Section 5) are involved in non-histaminergic itch pathways; the value of antihistamines in the treatment of wound itch is therefore uncertain. In view of the prominent role of inflammation in chronic wounds, one can speculate that dampening this with targeted anti-inflammatory agents could be of merit. Albeit there is no record of use of such agents for wound itch specifically, IL-31, IL-4, and IL-13 signaling is targetable through several commercially available agents with great success in other inflammatory states of skin; these include nemolizumab [255], dupilumab [256], lebrikizumab [257], tralokinumab [258], and JAK inhibitors [259]. Data are limited regarding the effect of immune suppression on malignant transformation—occasionally seen with chronic wounds—so monitoring is recommended [260]. Along those lines, inhibition of neuropeptides, for example with the Neurokinin-1 receptor (NK1R) antagonist aprepitant [261], might also be worth exploring. In cases where a neuropathic component is suspected, treatment with neuropathic agents can be considered in combination with anti-inflammatory and general itch-alleviating measures, with the added benefit of simultaneously addressing concomitant neuropathic pain.

Addressing background stasis dermatitis (SD) is important in the case of venous ulcers. There are no approved treatments for SD, but compression therapy is the mainstay [208,262]. Liberal moisturization is recommended, while Unna boots with topical corticosteroids can also be helpful [208]. In addition to the above, surgical intervention has been shown to expedite ulcer healing. Antibiotics and antiseptics are needed to address associated bacterial colonization or infection [208].

Reducing the wound bioburden is also a critical component of chronic wound therapy in other contexts [61]. In view of the potential contribution of microbes to wound-related itch, one can speculate that antibiotic therapy would contribute to the alleviation of associated itch through two distinct mechanisms: firstly, by preventing the release of pruritogens from microbes, and secondly, by reducing associated inflammation. Topical delivery of antimicrobials may be necessary in view of the resistance of biofilms to systemic antibiotics [29].

With regards to dressings, an international wound expert panel identified ideal characteristics to address itch, in particular ones that avoid allergens and sensitizers, along with optimizing vapour–moisture transmission rates to limit skin maceration and microbial proliferation [10]. Active dressings, such as films, hydrocolloids, hydrofibers, and foams, which provide a moist environment without sticking to the wound bed, can prevent dressing-related itchiness [263]. Chitosan-based hydrocolloid dressings (e.g., Tegasorb, 3M, Saint Paul, MN, USA) indeed led to a reduction in itching frequency over three weeks compared to the control group in a study of 80 subjects with refractory wounds [264]. Finally, itch in highly exudative wounds such as malignant wounds or hidradenitis can be limited with the use of absorbent dressings, including alginates, hydrofiber dressings, foams, and absorbent pads [265].

## 10. Conclusions and Future Directions

Despite evidence that itch is highly prevalent in healing wounds, there is a paucity of studies focusing on this issue, and our understanding of the mechanisms driving it remains limited. Further research is needed to accurately quantify the impact of chronic wound itch on patient quality of life, progress of healing, and healthcare resources. Robust clinical profiling of wounds of different etiologies, depths, and stages of healing is required to accurately capture the extent of the problem. Finally, further molecular studies can delineate cytokine and neuropeptide interactions with immune and neural cellular components, eventually leading to the development of more sophisticated therapeutic approaches. Importantly, the present review highlights the value of identifying and addressing itch as part of mainstream wound care.

## Figures and Tables

**Table 1:** Mediators of wound healing that are also known direct pruritogens.

Mediator	Role in Wound Healing	Role in Itch
CXCL10	Attracts immune cells to the injury site [74] Promotes wound re-epithelialization [75] Regulates fibroblast motility [76] Mediates neovessel regression during tissue remodeling [75]	Excites primary sensory neurons by binding to CXCR3 [77] CXCL10/CXCR3 signaling is involved in chronic itch [78]
IL-4 and IL-13	Suppress the initial inflammatory response for successful transition to the proliferative phase of healing [79] Promote phenotypic shift to M2 macrophages [79,80,81] Regulate ECM formation and remodeling [79,81,82]	Directly activate skin neurons involved in itch-sensory pathways [83] Sensitize DRG neurons to itch stimuli [83,84] Neuronal IL-4Rα signaling is necessary for chronic itch [83]
IL-31	Induces type I collagen production [85] Promotes inflammation through induction of chemotactic factors production [86] Promotes tissue remodeling through induction of MMP production [86]	Activates IL-31RA on a subset of TRPV1-positive DRG neurons, mediating T helper cell-dependent itch [83,87] Promotes neurogenic inflammation [88] Activates basophils to produce IL-4 and IL-13 [89] Mediates wound-induced itch responses [66]
Histamine	Accelerates wound healing through the action of basic fibroblast growth factor [90] Enhances keratinocyte-mediated wound healing and pathogen clearance functions primarily through HRH1 [91] Stimulates collagen synthesis by fibroblasts [92,93]	First recognized pruritogenic molecule [94] Mediates acute itch via activation of mechanically insensitive unmyelinated C-fibers in skin [95,96] in a TRPV1-dependent fashion [97] Upregulates the Th2 cell-attracting chemokine CCL18 in M2 macrophages [98]
Serotonin (5-HT)	Promotes chemotaxis during the inflammatory stage of wound healing [99,100] Accelerates keratinocyte and fibroblast migration [101] Promotes fibroblast proliferation and differentiation to myofibroblasts [101,102]	Mediates acute and chronic itch through HTR7, functionally coupled to TRPA1 [103] Facilitates GRP-GRPR dependent itch via 5-HT1A receptor [104,105] TRPV4 [106] and TRPC4 [107] are also linked with 5-HT-induced itch
Proteases	Tryptase Promotes epithelial cell migration and proliferation [108] Breaks down ECM [109] Increases fibroblast proliferation and collagen production [110,111]	Tryptase Induces pruritus via binding to PAR-2 receptors [112,113]
	Trypsin Enhances migration, adhesion, and proliferation of fibroblasts and macrophages via PAR-2 activation [114] Enhances collagen production [114] Potentiates monocyte differentiation to fibroblast-like cells (fibrocytes), accelerating wound healing [115]	Trypsin Induces pruritus via binding to PAR-2 receptors [116,117]
	Cathepsins Process mediators responsible for neutrophil chemotaxis during the inflammatory stage of wound healing [118] Contribute to ECM remodeling [119,120]	Cathepsins: shown to induce itch via PAR-2 activation in TRPV1-expressing DRG neurons [121,122] Mrgprs activation [123] Production of endothelin-1 in the epidermis [124]
Periostin	Promotes wound re-epithelialization [72] Stabilizes the ECM [125] Induces myofibroblast differentiation through TGF-β signaling and promotes wound contraction [40,125]	Secreted by keratinocytes in response to TSLP via the JAK/STAT pathway [73] Induces pruritus by binding to integrin αVβ3 on itch-transmitting DRG neurons [73] Stimulates immune cells to release IL-31 and other itch mediators [126]
Neuropeptides (including but not limited to substance P, CGRP, GRP, NMB, NPY)	Induce vasodilation and increased permeability [49,127] Regulate immune cell chemotaxis and function [49,127] Encourage keratinocyte and fibroblast migration and proliferation [49,127] Contribute to angiogenesis in the proliferative phase of wound healing [49,127] Influence ECM remodeling [49,127]	Neuropeptides released by peripheral sensory fibers drive neurogenic inflammation [128] Neuropeptides released by peripheral sensory fibers are involved in peripheral sensitization [68] Substance P stimulates immune cells to produce itch mediators IL-4, IL-13, histamine, and serotonin [129,130] GRP and NMB mediate itch at spinal level [131,132]
Lipid mediators	Leukotrienes Attract immune cells to the site of injury [133,134,135] Increase pro-inflammatory cytokine production [136] Promote collagen deposition [137]	Leukotrienes Initiate and perpetuate the inflammatory response in pruritic skin disorders [138] LTC4 elicits acute and chronic itch via binding to cysteinyl leukotriene receptor 2 (CysLT2) on DRG neurons [139] Keratinocyte-derived LTB4 mediates IL-31 and substance P-induced itch [140,141]
	LPA Attracts immune cells to the wound site [142,143] Promotes migration, differentiation, and proliferation of keratinocytes and fibroblasts [144] Promotes wound contraction [145] Participates in ECM remodeling [146]	LPA Pruritogen in cholestasis via activation of the LPA5 receptor [147,148,149]

Abbreviations: AD, atopic dermatitis; CGRP, calcitonin gene-related peptide; CXCL10, C-X-C motif chemokine 10; CXCR3, C-X-C motif chemokine receptor 3; CysLT2, cysteinyl leukotriene receptor 2; DRG, dorsal root ganglia; ECM, extracellular matrix; GRP; gastrin-releasing peptide; GRPR, gastrin-releasing peptide receptor; HRH, histamine receptor; HTR, hydroxytryptamine receptor; IL, interleukin; JAK, Janus kinase; LPA, lysophosphatidic acid; LT, leukotriene; Mrgpr; Mas-related G-protein-coupled receptor; NMB, neuromedin B; NPY, neuropeptide Y; PAF, platelet-activating factor; PAR, protein activated receptor; TGF, transforming growth factor; TRP; transient receptor potential; TSLP, thymic stromal lymphopoietin.

## Abbreviations

The following abbreviations are used in this manuscript:

AD	Atopic dermatitis
CGRP	Calcitonin gene-related peptide
CXCL10	C-X-C motif chemokine 10
CXCR3	C-X-C motif chemokine receptor 3
CVI	Chronic venous insufficiency
CysLT2	Cysteinyl leukotriene receptor 2
DEB	Dystrophic epidermolysis bullosa
DETC	Dendritic epidermal T cells
DRG	Dorsal root ganglia
EB	Epidermolysis bullosa
ECM	Extracellular matrix
GRP	Gastrin-releasing peptide
GRPR	Gastrin-releasing peptide receptor
HRH	Histamine receptor
HTR	Hydroxytryptamine receptor
IGF	Insulin growth factor
IL	Interleukin
JAK	Janus kinase
KGF	Keratinocyte growth factors
LPA	Lysophosphatidic acid
LT	Leukotriene
MMP	Matrix metalloproteinase
Mrgpr	Mas-related G-protein-coupled receptor
NGF	Nerve growth factor
NK1R	Neurokinin-1 receptor
NMB	Neuromedin B
NPY	Neuropeptide Y
NRS	Numerical rating scale
PAF	Platelet-activating factor
PAR	Protein activated receptor
QoL	Quality of life
ROS	Reactive oxygen species
SD	Stasis dermatitis
SP	Substance P
TGF	Transforming growth factor
TRP	Transient receptor potential
TSLP	Thymic stromal lymphopoietin
